# What does the Thinking about Relationalism and Humanness in African Philosophy imply for Different Modes of Being Present in the Metaverse?

**DOI:** 10.1007/s11948-024-00496-y

**Published:** 2024-07-23

**Authors:** Cornelius Ewuoso

**Affiliations:** Steve Biko Center for Bioethics, University of Witwatersrand, Johannesburg, South Africa

**Keywords:** Metaverse, Global south, Relationalism, Humanness, Ways of being present, African philosophy

## Abstract

In this article, I interrogate whether the deployment and development of the Metaverse should take into account African values and modes of knowing to foster the uptake of this hyped technology in Africa. Specifically, I draw on the moral norms arising from the components of communal interactions and humanness in Afro-communitarianism to contend that the deployment of the Metaverse and its development ought to reflect core African moral values to foster its uptake in the region. To adequately align the Metaverse with African core values and thus foster its uptake among Africans, significant technological advancement that makes simulating genuine human experiences possible must occur. Additionally, it would be necessary for the developers and deployers to ensure that higher forms of spiritual activities can be had in the Metaverse to foster its uptake in Africa. Finally, I justify why the preceding points do not necessarily imply that the Metaverse will have a higher moral status than real life on the moral scale that can be grounded in Afro-communitarianism.

## Introduction

Perspectives from scholars in Computer Science, Engineering, Business and Sociology mostly dominate the emerging literature on how the Metaverse ought to be *deployed*. Additionally, existing contributions to the ethical discourse on the *development* of the Metaverse mostly reflect the knowledge systems, values, ways of being and modes of encountering the world dominant in the Global North. Other ethical reflections, particularly from the Global South, are required to foster its uptake in the Global South. Studies show that individuals will likely utilize or accept projects that align with their values and knowledge systems. Value and knowledge systems’ alignment influence behaviours (Campos-Mercade, 2021) and enhance collaborations (Macedo & Camarinha-Matos, 2017), while non-value (or non-knowledge) systems’ alignment can cause an organization to fail to reach its potential (James, 2014), or effect positive changes (Branson, 2008). The non-alignment with African values and knowledge systems can undermine the uptake of the Metaverse in Africa. For this reason, it is vital that the principles and values that underlie the Metaverse’s development, as well as its deployment, are sensitive to the culture and context-specific modes of knowing or encountering the world in regions where it hopes to gain acceptance.

To address questions around fostering the uptake of the Metaverse in Africa, this article draws on the moral norms arising from the components of communal interactions and humanness in the scholarship of Afro-communitarians (or henceforth, African moral philosophers) to describe how the Metaverse’s development, as well as its deployment, can (better) reflect African values. What (more) is required? The reader should observe that this article does not defend the position that the thinking about the components of communal interactions and humanness emanating from the works of the African philosophers it identifies is the most *philosophically sound for this task*. Instead, the goal of this article is primarily evaluative. Precisely, the article draws on these accounts to interrogate the question, “Should the deployment of the Metaverse, as well as its development (and its underlying principle), take into account African values and modes of knowing to foster the uptake of this hyped technology in Africa?” To address this question, the article draws on the moral norm arising from the components of these key values (communal interactions and humaneness) grounded in African moral philosophy to contend the following points: (i) the deployment of the Metaverse and its development ought to reflect the core African moral vales/principles, (ii) to adequately align the Metaverse with African core values, and thus, foster its uptake among Africans, significant technological advancement that makes simulating *genuine human experiences* possible must occur, (iii) in addition to (ii), it would also be necessary for the developers and deployers to ensure that higher forms of spiritual activities can be had in the Metaverse to foster its uptake in Africa, (iv) to better reflect African values and foster its uptake in Africa, certain modes of being present, particularly body swapping ought to be made impermissible in the Metaverse, and finally (v) Suppose (ii), (iii) and (iv) have been satisfied, the Metaverse will still have a lower moral status than real life. While (v) does not necessarily imply that many Africans will find the Metaverse unacceptable, it does prove that the Metaverse will not rank higher than the real life on their moral scale. The article also considers objections that contend that the development and deployment of the Metaverse can ever align with African values given that, (i) the risks associated with the Metaverse far outweigh its benefits and, (ii) the cost of running the Metaverse will come at significant harm to the environment, something many Africans tend to have special relations with.

The article’s approach should interest academic scholars, at least for its own sake and its instrumental value. Particularly, there are ethical accounts of the Metaverse. For example, studies like the publication by Anshari et al. (2022) have interrogated core ideas about permissible behaviours in the Metaverse by drawing on moral approaches dominant in the Global North. However, no one that the author knows has contributed to the ethical discourse on the Metaverse by drawing on the two concepts it draws on. Notice that there are published studies on the ethics of technology broadly from an African philosophical perspective. For example, McStay (2022) has explored whether empathy can be computed—but mostly in relation to chatbots and vector-based properties—from the perspective of Ubuntu philosophy. Equally, Friedman (2022) has interrogated ethical concerns around replacing human relations with humanoid robots from the positionality of African humanism. Also, Morgan (2021) has drawn on virtues like solidarity in African philosophy to *espouse virtual communitarianism (*a new moral framework). In addition, Ikhane (2020) has drawn on *symontosis* grounded in African philosophy to explore how virtual reality impacts knowledge production and shapes lived reality. Finally, Bekele et al. (2023) have also drawn on African epistemologies to explain how (immersive) technologies can be integrated into African education. However, the specific ways the moral norms arising from the components of communal interactions and humanness in African moral philosophy can inform the Metaverse’s development and deployment to foster its uptake in Africa has not been interrogated to any significant degree, if at all. This article contributes towards filling this gap.

## Conceptual Clarification

This section attempts a brief conceptual clarification of the Metaverse and a broad description of the African moral philosophy it draws.

### Understanding the Metaverse

The Metaverse deserves to be unpacked. Admittedly, the Metaverse is a fascinating speculative, hyped, and nascent technology anticipated to succeed the internet. It is called by many names, including Web 3.0 and Future Internet. The term occurred first in Neal Stephenson's 1992 science fiction novel Snow Crash, wherein the author described a virtual world where users could escape and interact with one another through their avatars (Ifdil et al., 2022). Two examples of this digital world include World of WarCraft and Ready Player One (Spence, 2008). It is essential to observe that an actual Metaverse does not yet exist. What it will look like or how the technology will evolve are largely unknown. Nonetheless, some of its *anticipated* features are worth describing. It is a mixed reality (sometimes called extended reality), combining physical and digital platforms. For this combination, the Metaverse leverages virtual and augmented realities technologies to enable a socially focused 3D virtual multi-users space that is perpetual, persistent, scalable, autonomous, runs on blockchain and overcomes many limitations of the natural world (Skalidis et al., 2022b). Both virtual and augmented reality are differentiated by the amount of the physical world included in the digital experience, with virtual reality at the end of the spectrum of the digital experience. "[Augmented realities are] real-time interactive experiences of a real-world environment through digitally generated three-dimensional representations integrated into real-world stimuli and existing reality]" (Wu & Ho, 2022). As an augmented reality, creators can superimpose additional information on the physical world by adding a digital environment to the physical one. Virtual reality is distinct from augmented reality and offers a more immersive experience than augmented reality.

### Describing African Moral Philosophy

The African moral philosophy the article draws on also deserves to be described. It is common to call African moral philosophy a relational philosophy (Ewuoso & Hall, 2019). Whilst this is true of African moral philosophy, a moral theory does not need to be African to be relational (Metz, 2013). There are also relational theories outside Africa. Examples of non-African scholars who have developed a *corpus* of work on relationalism include Alasdair Macintyre, Amitai Etzioni and many Care Ethicists in the West. Similar to African scholars like Metz, Jonathan Chimakonam and Kevin Behrens, this article holds that a relational approach is African to the extent that prominent values on the continent inform it in a way that is typically not found elsewhere. For this reason, Chimakonam and Ogbonnaya (2022) define African moral philosophy as “an African culture-inspired communal living model that stresses shared, mutual…interdependence and complementarity…relationship, togetherness, co-existence, co-dependence, and co-responsibility toward the community and others.” This appears to be the opinion of Innocent Asouzu, Julius Nyerere, Chris Ijioma, Godfrey Ozumba, Leopold Senghor and Kwame Gyekye. In Gyekye's (1995) view, features of modes of being and ways of life are sufficiently common on the continent that could usefully inform our thinking about an African moral system. This article does not claim that all Africans share these values. It is possible to find differences in Africa regarding what values are essential and should be given *any* significance. This article aims to identify the common ones, particularly communal interactions and humanness, describe moral norms that can arise from their components, and finally, draw on these norms to interrogate the question it raises (Bekele et al., 2023; Ewuoso et al., 2022).

## Is the Metaverse Intrinsically Problematic from an African Perspective?

To address this article's core question, this section makes the case that the deployment of the Metaverse and its development ought to reflect the core African values. A failure to reflect its core values can undermine the Metaverse’s uptake in Africa. Here, the article demonstrates that the Metaverse is not intrinsically problematic since it already fosters these values in many ways. On this account, I agree with Morgan (2021) that hyped technologies that incorporate virtual reality technologies like the Metaverse can and sometimes do reflect African values. To understand how, it is important to reiterate that African moral philosophy tends to entail the idea of interaction, particularly communal interaction, interconnection and the requirement to see oneself through others—or exhibit other-regarding behaviours (Chigangaidze et al., 2022). The dominant position in this scholarship is that Africans are communitarian or interactive by nature. On this account, the right action is one that enhances human connection and relations. Precisely, an action is immoral (in this philosophy) to the extent that it causes division (Chimakonam & Ogbonnaya, 2022; Ewuoso & Hall, 2019). Communal interactions have moral implications for many Africans. Precisely, *a person is a person through other persons.* As Mfutso-Bengo and Masiye (2011) remark, "To be, is to belong to each other: no man is an island…" Equally, communal interaction is a fundamental organizing factor in society. Individuals with resources are required to render assistance and support those less resourced since one's misfortune is everyone's misfortune. The choices we make can advance other people's lives or end them. As Kayange (2018) observes of the Bantu people, "Bantu individual is communitarian in his/her way of thinking and life."

The communal relationship also has moral implications for what goal should inform the development and deployment of the Metaverse. This should aim to connect rather than divide individuals. Admittedly, the Metaverse already fulfils the African moral requirement to connect, rather than divide, individuals. Notably, although still a hyped technology, the Metaverse aims to replicate, extend real-life relationships and activities in digital forms (Ifdil et al., 2022). This is not necessarily problematic from the point of view of the philosophy that emphasizes connections and relations. Precisely, the Metaverse will be a world of—among other things—endless relational possibilities where our imagination is the only limit. In the Metaverse, individuals will enjoy the freedom to engage, socially interact, play games with one another, conduct business, and telework. They will be free to perform and undertake relational tasks in (more enhanced) ways, attend concerts, receive lectures, have financial interactions, travel, make clinical appointments, learn in 3D, trade digital assets, or live their daily lives through avatars. To realize this goal, the Metaverse is open (common, easily accessible and artificially co-created and decentralized) and interactive. Unlike Web 2.0, where users do not participate fully in generating contents, the developers of the Metaverse aim to give users the ability to edit, control, and own the contents of their experience, thus, giving them more benefits of the digital world by blurring the power asymmetry between companies and users and equalizing relations amongst users (Benjamins et al., 2022). For example, the South Korean social media ZEPETO allows users to edit their avatars by giving the same features they prefer and in the process, create a virtual identity which could then be used to interact, trade with other human-controlled avatars/digital objects, or attend events (Zhang et al., 2022). Customizing avatars may enable capabilities that users lack in the real world. For example, a person with a disability who, as a result, is home-bound could edit their virtual twin to move around and perhaps walk or have other expressions of movement that they lack in the real world. Gaining such expressions of movement could enhance their capacity to bond and relate more with others.

Suppose the Metaverse aims to bring people together in the digital space and not to divide them. Suppose the Metaverse is not merely a get-away but a place where users can exist, live their lives in unique ways, work and have social interactions that are unconstrained by space, location and time of the natural world (Petrigna & Musumeci, 2022). In that case, this technology aligns with the moral theory that emphasizes social connection rather than division. From this perspective, the technology is not *inherently* problematic.

### Communal Interaction and the Ways of Being in Metaverse

This section contends that to adequately align this new technology (the Metaverse) with the core African values, and thus, foster its uptake among Africans, significant technological advancement that makes *genuine human experiences* possible must occur. Currently, difficulties with reproducing genuine human experiences persist. As the section demonstrates, this has implications for the psychosocial aspect of communal interactions. Precisely, given these difficulties, the psychosocial aspect of communal interactions *will likely not be* possible in the Metaverse when deployed unless there is a significant technological advancement. The section also justifies permissible activities in the Metaverse from the point of view of the value this section draws on.

It is important to state from the outset that though the account of interaction that the article provides consists of different components, one does not necessarily honour norms of interaction by honouring *just one* of these core components in the same way one does not breach these norms by failing in one core component. Notably, these components are not binary such that one needs to fulfil all core components to honour communal interaction. Similarly, each component elicits different duties, and one does not violate the overriding norm elicited by each component through a failure to honour one or two of these duties.

First, in the scholarship of African moral philosophers, communal interaction is a scalar conception whereby fulfilling more components of this interaction constitutes a higher level of other-regarding behaviours (Ewuoso & Hall, 2019; Gade, 2011). Second, communal interaction ought to be prized and *not maximized* (Metz, 2021; Morgan, 2021). For example, Utilitarians contend that we ought to maximize overall well-being. On their account, it is not necessarily immoral to harm an individual if it maximizes well-being. Contrarily, in Metz (2021) deontological reading of African moral philosophy, the means of promoting communal interaction are equally important. Suppose the harm of promoting interaction outweighs the benefits of promoting the same. In that case, it ought not to be performed. Yet African moral philosophy differs from deontological theories like Kantianism since it emphasizes social cohesion, rather than autonomous capacity, as the core of morality.

A key component of communal interaction is its psychosocial aspect. The psychosocial aspect of communal interaction tends to entail the combination of identification and goodwill (Metz, 2007). Harmony is another word Metz (2007) uses for communal interaction that this article describes. Thus, harmony consists of a combination of identification and goodwill. Individuals identify with each other when they emotionally (for example, through higher forms of sexual intimacies), behaviourally, and socially share a way of life. As Metz (2007) clarifies, there is a relationship of goodwill when we wish others well, aim to enhance their quality of life for their own sake and rejoice when they flourish or agonize when they are harmed. In this regard, psychosocial aspects of communal relationships involve positive, transparent and genuine behaviours or affective emotions towards one another. Negative emotions and ill-will would be anti-communal. Some scholars point out that the psychosocial aspect of communal interaction was a critical factor—not the *sole factor*—in lowering COVID-19 infection/death rates in Africa (Ajei, 2022; Chimakonam & Ogbonnaya, 2022). Precisely, many Africans did not object to restrictive measures that limited their freedom imposed by different governments because this is what a sensitive communal being will do for the greater good of public health and safety.

It is essential to point out that while some forms of restrictive measures were accepted, some scholars still think that isolation during the COVID-19 pandemic fails to realize the ideals of communal interaction. Precisely, isolation entails the idea that we can exist alone and be separated from others. It (isolation) undermines the capacity to exhibit caring behaviours amongst relatives. In some studies, participants—reflecting on isolation from the African communal perspective—claimed that isolation created anxiety, disconnection, loneliness and moral anguish, caused mental instability and compromised trust relations in clinical care (Kainja et al., 2022). Isolation prevents individuals from enjoying the feeling of *being there*.

The challenge for the developers of the Metaverse will be to demonstrate that positive, transparent and genuine (or deep) psychosocial human behaviours can be had and reciprocated, particularly, by non-human avatars in the Metaverse. Equally, can higher forms of this psychosocial aspect, like sexual intimacy, be genuinely simulated, digitized or had by human users and visitors in the Metaverse? On the one hand, this question is important to align the deployment of the Metaverse with this African value. On the other hand, this question is also vital since ideological/philosophical, societal and economic factors motivate or power the Metaverse: its development and deployment (Xi et al., 2022). For example, the Metaverse is influenced by philosophical beliefs that the world/life can be simulated. To understand how mathematical and informational structures, scholars believe, underline reality. Reality derives its significance from these structures. “Reality and even consciousness itself has substrate-independence, or substrate-neutrality, meaning that complex phenomena such as consciousness are not contingent on stuff a system is made of” (McStay, 2022).

Although different conceptual beliefs about simulation exist, empirical evidence is unclear on what aspects of reality could be simulated or digitized, and whether such simulations are genuine. For example, participants in one study claimed that their best effort to simulate psychosocial relationships with their sick loved ones through video calls left them even more frustrated (Mulaudzi et al., 2022). Friedman (2022) echoed the views of these participants when they remarked that non-humans cannot genuinely reciprocate human sentiments. In the Metaverse, we may come to like non-human controlled avatars or care for them. But this relationship will be a unidirectional emotional bond. Non-human controlled virtual objects, and avatars would be unable to reciprocate—but could simulate—this behaviour. Genuine human sentiments can only be experienced and expressed by humans. Nonetheless, some participants in a study on tourism believe that virtual tourism has similar effects on them as *actual* tourism (Rahman & Bhowal, 2017).

Though the thinking about the psychosocial aspect of communal interactions in African moral philosophy does not imply that these forms of interactions ought not to be simulated, the expressed views regarding simulation still point to the difficulty of genuinely replicating this psychosocial component by current technologies. Future (technological) advancements should aim to make positive, transparent, and genuine psychosocial aspects of communal interactions possible to better align the Metaverse with African values. Precisely, with further progress, future computers may be capable of more complex experiences of what we accept to be intrinsic to humans. With enough computing powers, reality, consciousness, and the human mind could be perfectly replicated. This will positively impact the uptake of this technology in Africa. Some advanced online games have successfully reproduced human facial expressions in avatars (Tan et al., 2022). Sights and hearing have also already been rendered possible by certain VR technologies. Equally, specific user interfaces like the haptic technologies already enable one to enjoy a sense of touch to some degree, implying that a range of typical human experiences may be possible in the Metaverse through human-controlled avatars. To replicate real-life experiences, enable individuals to increase their sense of self, increase their feeling of presence—or produce genuine emotional, social or behavioural reactions—in the Metaverse, future developments in technologies that enable taste and smell will also be required.

Could more advanced forms of these technologies make it possible to have even more advanced forms of psychosocial experience in the Metaverse? There is evidence that some digital technologies can produce basic emotional reactions like joy, empathy, compassion, sadness, gratitude or awe, some of which are essential for having and enjoying more advanced or deep forms of the psychosocial component of communal interaction like sexual intimacies. Replika, Microsoft’s Xiaoice, SimSimi, and Woebot are a few chatbots that claim to be sympathetic and compassionate AI tools (McStay, 2022). It might even become possible to print out one’s taste or smell in the future (Benjamins et al., 2022).

### Simulating Spiritual Experiences

Although having positive, transparent and genuine psychosocial human behaviours in the Metaverse (when deployed) is essential, this is not sufficient. This section contends that in addition to these, it would also be necessary for the developers and deployers to ensure that higher forms of spiritual activities can be had in the Metaverse to foster its uptake in Africa. Notably, communal interaction also has a spiritual component; the relationship is not only with visible entities but also includes interaction with spiritual entities, extending to dead ancestors, spirits and deities. This is the view of Ramose (1999), who asserts—through his onto-triadic conception of being—a three-dimension of relatedness (with the dead, living and yet born) of a human being. These dimensions are essential to understanding being in Africa. One study exploring the importance of communal relationships in mental health found that many participants believed that mental illness has a spiritual aspect to it and is caused by evil spirits or witchcraft (Kainja et al., 2022). The Spiritual and physical worlds are fundamentally related and interrelated. For this reason, Forster (2010) reckons that "the unity and harmony of personhood expressed in [African Ubuntu philosophy] stretches from the world seen through the naked eye to the world of ancestors, the spirit world." Although this article discusses key components of humanness in the subsequent section, notice that humanness also requires developing the spiritual side of oneself. In this regard, illnesses should be treated with natural and spiritual remedies since they have both natural and spiritual causes. For example, some scholars attribute the HIV/AIDS scourge in South Africa to the failure to relate/honor the ancestors through rituals (Cordeiro-Rodrigues & Metz, 2021). The reader should notice that this article does not necessarily believe that HIV/AIDS scourge in South Africa has a spiritual aetiology since it is, in fact, an infection, and the scourge has a scientific explanation (Goncalves, 1994).

Some spiritual experiences may be possible in the Metaverse, implying that the Metaverse could reasonably offer individuals some opportunities to enjoy (limited) spiritual activities. For example, individuals could hold festivals, attend a spiritual gathering like a church service, or consult with their spiritual leader like a priest or imam (Afriyie, 2023). However, I am not aware of any technology that purports to one day be able to simulate more profound spiritual experiences like authentically communicating with spirits. Although extended reality technologies are required to access Metaverse, what is extended is the natural environment. The devices like head-mounted displays that make this possible can only capture (large) information or data about users like users' position and movements, observe their direction, and/or scan and monitor users' immediate natural environment *or the coverage zone of the device* (Fernandez & Hui, 2022). Devices that can capture transcendental realities cherished by Africans do not yet exist. Some key advancements in technologies, as well as ambitious projects, to enable the Metaverse are worth mentioning here. For example, NVIDIA's goal of a photorealistic image is worth mentioning. Additionally, the construction of virtual hospitals is underway to deliver digital health care to often difficult-to-reach population groups (Skalidis et al., 2022a). Future advancements in technology may make transcendental experience possible in the Metaverse.

The point here is that the technologies for having more intimate forms of spiritual experiences like communication with ancestors and relating with spirits do not yet exist, to the best of this author’s knowledge. This will be another challenge for the developers of the Metaverse. Suppose the creators, designers and deployers want to align the Metaverse perfectly with the thinking about communal interactions in African moral philosophy. In that case, investment in technologies that can make the spiritual component of communal relationships possible will be required. The growing interest in the Metaverse could one day make this possible. Precisely, the Metaverse continues to be powered by financial and strategic investments by governments and standards organizations like the Metaverse Standards Forum, as well as companies and big players in the tech industries like Mark Zuckerberg, Apple, and NVIDIA (McStay, 2022). For example, in 2021, Mark Zuckerberg changed Facebook's name to *Meta,* indicating that the company will subsequently be metaverse-first (McStay, 2022). As the interest in the Metaverse increases and new technologies are developed, a higher degree of spiritual component of communal relationships may become possible. *More cynically*, we may one day be able to speak with a *spirit-controlled*—and not an AI-controlled—avatar of one's dead ancestor in the Metaverse.

### Humanness, African Philosophy and Body Swapping

The Metaverse faces another difficulty that can undermine its uptake in Africa. Notably, to better reflect African values and foster its uptake in Africa, this section draws on the thinking about humanness to claim that certain modes of being present, particularly body swapping, should be made impermissible in the Metaverse. Humanness in the scholarship of African philosophers tends to incorporate moral, ontological and biological components. Notably, the thinking about humanness is sometimes moralized and adequately captured in the concept, *Ubuntu.* Literarily, *Ubuntu* entails the moral duty to exhibit certain virtues like compassion, hospitality, reciprocity, and honesty, to name a few. It is an invitation to empathize, sympathize and show solidarity. *Your misfortune* is my misfortune, and your successes are my successes (Chimakonam & Ogbonnaya, 2022).

The preceding paragraph implies that moral thinking about humanness is methodological (Chimakonam & Ogbonnaya, 2022). It describes how humanness is realized and/or affirmed. Notably, humanness is expressed within the actual context of relationships. In this way, communal relationships are related to humanness as the means of developing the latter. One is more or less of a human within the context of relationships. For this reason, Tutu (1999) retorts that humanness is contingent on belongingness and interaction. Hence the maxim *I am because we are.*

The moral and ontological components are intimately linked. In many accounts, the individual is an ontological being who can realize their beingness by normatively moving towards *human* welfare (Ewuoso & Hall, 2019; Gade, 2011). What makes an entity intrinsically worthy of our duties is its relational-ness. In this regard, an entity's failure/refusal to be moved towards others entails an act against its communal nature.

The ontological component necessitates the psychosocial component of *communal relationships*. Concretely, we accept to make some sacrifices, voluntarily limit our freedom to advance public health and stop the spread of [say] a virus since this is how a profoundly relational being will act. In other words, it aligns with my relational nature not only to act for your well-being but also to know as much as possible about you, your needs, capacities and weaknesses to *genuinely* fulfil the requirement of the psychosocial component of communal interactions or relationships.

Although the article alluded to genuine emotions in the previous section, what it considers genuine relationships should be described at some length. Genuine relationships imply relating in relevant ways and do so compassionately, respectfully and transparently. Against this background, one's capacity to genuinely interact with others is undermined when the terms of this interaction are unclear or when one does not have the material information to make informed decisions about such engagement.

The importance of genuine relationships or interaction in the ontological component of humanness will imply that certain ways of being present and showing up in the Metaverse will be impermissible, at least from this positionality. To understand how, notice that the Metaverse is *not* built or developed by one technology but a significant innovation and evolution of a blend of many technologies (including high-speed connectivity or 5th or 6th-generation telecomputers/network infrastructures) that will make certain capabilities possible in the Metaverse. For example, when the Metaverse is deployed, it will integrate social media. But it is not social media. Unlike the current social media platforms where interactions can be had in 2D or *a persona* created for the social media, in the Metaverse, interactions will be had in 3D, and *a completely new person or body swap*—that is different from the user—could be created for the Metaverse (Benrimoh et al., 2022). Body swap—within the context of the ethical discussion on the Metaverse—entails using an avatar that conceals the real identity of its human user (Riva & Wiederhold, 2022). The capacity to create an entirely new person for the Metaverse could make it easy to conceal one's real identity and, in the process, fail to interact genuinely. Here, it is important to clarify that although body swapping can be realized through the creation of an avatar (for the Metaverse) that conceals the *real identity* of the human user behind it, not all avatars in the Metaverse would necessarily have been body-swapped. Some avatars may reflect the real identity of the human user behind them.

Suppose the ontological component requires transparency in relationships. In that case, concealing one’s identity is a failure to be transparent. The ontological component of humanness has another implication for body swapping and identity concealment. Notably, body swapping is also a failure to wish *some* well. To understand how, the reader should note that the possibility of creating a new person for the Metaverse could cause the dilemma of dissonance. This dilemma occurs when the user emerges in the physical environment only to realize a mismatch of identities—between the digital and physical worlds. As Benrimoh et al. (2022) observe, the dissonance will be created when [users] emerge into the real world and remember that their real bodies cannot be altered with the same ease [as the body in the Metaverse}.” The dissonance *could* cause them to have a negative image of their real-life body (Fardouly & Vartanian, 2016). As the article demonstrates in a subsequent section, the real life has a higher moral significance than digital life in African moral philosophy. The impact of dissonance on individuals with different self-esteem issues like eating disorders will be huge (Benrimoh et al., 2022). The preceding indicates that the virtual life of an avatar could impact the user's real life. In fact, the long-term goal of the proponents is to develop the Metaverse in ways that there will be no borders (between virtual and real-life existences), and users may become so immersed in the environment that their *actual or real lives* become *nearly* indistinguishable from their virtual lives (Ifdil et al., 2022; Ikhane, 2020). For example, turning on my coffee machine in the Metaverse will have a similar effect on the coffee machine in my apartment. Both worlds will *be connected* through digital twins. Digital twins are mixed reality objects created to represent physical objects (behaviourally and in appearance). In the Metaverse, digital twins will allow users to enjoy a sense of presence or being there—through place illusion and perceived authenticity of other virtual objects and ourselves. The impact of place illusion has been negatively described in the literature. Specifically, individuals may find it challenging to disengage from the Metaverse. Those who manage to disengage from the platform may experience anxiety or the need to reconnect. The need to reconnect is further reinforced with the experience of dissonance. This risk is heightened in adolescents, youths and individuals who struggle with self-control (Benjamins et al., 2022). Some virtual games and social media platforms already have this effect on certain individuals. There are real risks to users' mental and physical health from spending a period in the Metaverse (Dunleavy et al., 2009; Weidinger et al., 2018). In fact, online platforms have been found to generate Zoom Fatigue by studies (Wiederhold, 2020) and exert significant pressure on individuals' mental health, sometimes causing depression, addiction and paranoid ideation (Xi et al., 2022). Suppose it is true that body swapping can cause dissonance. In that case, under the preceding assumption, body swapping can reasonably be adjudged to be a failure to wish, at least, individuals who experience dissonance well. From the African philosophical perspective that requires us to act in ways that will likely improve the life quality of others, an important way of wishing individuals well—within this context—will be to limit their exposure to or prohibit activites that will increase their likelihood of experieicing dissonance.

To accommodate this transformative technology's capability for body swapping, someone may counsel that African moral philosophers need to radically shift/alter their worldviews. Equally, African philosophers could also address this by de-emphasizing the requirement of transparency in interactions to accommodate the possibility that the avatar one relates with in the Metaverse may be different from the user behind it. It could also be an AI-controlled avatar.

In response, it does not seem right that African values should be adjusted to suit the Metaverse. Instead, the Metaverse must be more sensitive to people's ways of experiencing the world for the technology to be acceptable to them. The implication of the value of transparent/genuine interaction for the Metaverse is that it would be impermissible for users to construct a digital identity that is different from their real-life identity, even if others in the Metaverse were informed of this mismatch. It would be impermissible for them to *significantly* edit their avatar's features and properties or manipulate the same however they like to substantially change how they show up or conceal their real-life identity in the digital world. Such substantial change will create a false appearance that undermines the thinking about humanness in African moral philosophy. The moral norm that can arise from the ontological component of humanness—in African scholarship would imply that interactions between avatars ought to be genuine. Transparency enhances genuineness and is realized when the user's identity aligns with the avatar's identity. It would also imply that deep fakes will be impermissible in the Metaverse. As Morgan (2021) rightly point out, through their virtual communitarianism, trust and solidarity will develop when there is transparency and genuineness.

A critic may also contend that it is unclear why the phenomenon of body swapping is more problematic from an African philosophical perspective than any other perspective. In fact, philosophical approaches like Kantian ethics might consider body swapping morally concerning suppose it undermines autonomous capacity. In response, this article does not contend that body swapping is only (or more) problematic from the African moral philosophical perspective. While the article acknowledges that body swapping might be problematic from other perspectives, its primary concern in this section has been to provide African-inspired reasons why it is problematic from that positionality.

### Biological Component of Humanness and Simulation

Suppose body swapping and impermissible modes of being present are excluded, higher ways of genuinely relating and forms of spiritual activities can be enabled in the Metaverse. In that case, this section draws on the biological component of humanness to contend that the Metaverse will still have a lower moral status than real life.

While maintaining an existence in the Metaverse may not necessarily be unacceptable to Africans (suppose all the identified challenges were successfully addressed), real-life will have a higher moral significance and ought to be prioritized since this is where one enjoys authenticity. The preceding provides a chance to lay bare some of the ontological commitments taken for granted by the developers and deployers of the Metaverse. There is a *real* world in which we exist and *should* form the basis of our emotional, physical and psychosocial *beingness.*

To justify the preceding, it is worth pointing out that humanness also has a biological component. To understand how, note that there are different schools in African moral philosophy. One school is moralized form of African moral philosophy that contends that personhood and moral status are on actual participation in communal life. This is the view of Masolo (2010), who once affirmed that individuals are “‘…born humans but become persons.” This view has also been endorsed by Wiredu (1992) and Tangwa (2010), to name a few. A second school of thought is the modal African moral philosophy. This second school is particularly relevant for justifying my point. It contends that the capacity for, rather than actual, participation in communal life is required for moral status. Additionally, modal African moral philosophy sometimes uses the biological component of humanness to explain the idea of moral status. Specifically, only entities that can interact—and/or be interacted—with, and *in a way humans can*, have a moral status. This conception of moral status ought to be unpacked. First, to have moral status is to be an object of direct duties (Cordeiro-Rodrigues & Ewuoso, 2021b). Entities that do not have moral status, as Metz (2012) clarifies, are genetically/biologically unable to fulfil the psychosocial component of communal interactions. They can neither share a way of life with others nor be communed with. An example is a stone. This African conception of moral status shares features similar to care ethics, particularly Nel Nodding's conception of the same. But it also differs from it. Both conceptions agree that individuals who can interact with others have some moral status. However, For Noddings (1984, 1988), entities that cannot commune lack this status. In contrast, in the African conception, the absence of the capacity to commune does not necessarily imply that the entity lacks a moral status.

Moral status is also held in degrees amongst entities that fulfil the biological condition (Bekele et al., 2023). The higher the capacity for communal relationship, that is, to commune and be communed with, the higher the moral status. Typically, adult humans—though this is not always the case—meet the requirement for higher moral status. Therefore, they are more morally considerable than entities with a lower moral status, like infants, embryos and animals (Metz, 2012). To understand how, recall that the moralized conception of personhood holds that personhood is not given at birth but acquired over time. The older one becomes, the more personhood one develops and the more intricately connected to the community one becomes. The more intricately connected to the community, the greater the damage that could result from one's death. Thus, the death of older adults tends to cause more pain for many in different African communities (Cordeiro-Rodrigues & Ewuoso, 2021b).

Second, *in a way humans can* implies that humanness requires genetic features, notably a body. This has many implications for how we think about the moral status of, say, Sophia, who became the first robot to be afforded citizenship in Saudi Arabia (Parviainen & Coeckelbergh, 2021), or the moral status of both human-controlled and non-human-controlled avatars in the Metaverse.

The preceding concern about the moral status of avatars deserves to be unpacked. First, there is a question about simulation itself. Although the article discussed simulation in a previous section, it is still vital to differentiate between simulating emotions and producing the same. Non-human controlled avatars (and humanoids and other virtual objects) *may succeed* in simulating compassion, empathy and emotions. This is not the same as producing them. Against this background, they will have a lower moral status even when human-controlled avatars erroneously believe these virtual objects to be human-controlled. This is because these virtual objects—as subjects of relationships—will likely be dealing in fake emotions and compassion that are not authentic. In this way, they are less likely to *genuinely share a way of life with other human-controlled avatars*. The key here is identification or genuinely sharing a way of life with others. Individuals and entities who fail to share a way of life with others genuinely will have lower moral status even when others can share a way of life with them.

One counter-response to the preceding is the Theory-theory account of emotions. The theory-theory account of emotions describes emotions as “inner [states with] law-like effect” (Eickers & Prinz, 2020). For example, when we see someone cry, we could reasonably infer that they are sad because of a law that says sadness is an inner state that could cause one to cry. On this account, what matters is that, i) emotions are exhibited, and ii) exhibited emotions are linked to an inner state as their root cause. Within this context, suppose machines exhibit certain emotions that we normally believe are rooted in an inner state. Equally, suppose humans cannot tell the difference between a simulated emotion and real emotion. In that case, we can call it emotion, implying that non-human-controlled avatars will have similar moral status as humans and their avatars (McStay, 2022). Herein, what would matter is whether these virtual objects can exhibit human-like emotions at all and convincingly. In response, the motives and interests of producing empathy, compassion and emotions—at least from the African moral philosophy perspective—also matter. Notably, one is more human due to these acts (McStay, 2022). It seems intuitive that non-human controlled virtual objects can hardly have *the intention to be* more human through their actions, and this undermines their moral status.

But is it possible to simulate humanness itself? Higher forms of becoming a person require the biological component. As Metz (2010) argues, "a person could have the ability to engage in loving or friendly relationships if she were a purely *physical creature*, and so this view is independent of any spiritual notion that a person's dignity is a function of God." Biological relatedness is essential for personhood, humanness and higher moral status. Otherwise, chimpanzees who have some of our DNA would be human. Avatars may *look like* humans but will not have human DNA.

Humanness also requires and develops through physical touch, physical presence, and intimacy, in addition to acting in relevant ways. As the article pointed out in the previous section, more advanced haptic technologies could enable higher levels of these experiences. Nonetheless, it is doubtful that they can replace physical experiences or more intimate forms of physical experiences like sexual relations. In fact, online relationships tend to be considered less meaningful in comparison with physical ones (Cordeiro-Rodrigues & Ewuoso, 2021a). Similarly, one may prepare and eat food in the Metaverse. However, this may trigger the hunger hormone (ghrelin) in real life, thus failing to result in satiety.

The significance of the preceding is that engagements (such as helping each other out) between human-controlled avatars in the Metaverse will have a lower moral status than similar engagements between humans in real life, given the importance of physical presence and intimacy for according higher moral status. It also implies that engagements (like conducting business) between a human-controlled avatar and a non-human-controlled avatar will have a lower moral status than a similar engagement between two human-controlled avatars, given the importance of genuineness and transparency of relationships. Furthermore, the *inability to enjoy higher forms of physical experiences in the Metaverse implies that avatars will not have a moral status similar to that of* their users. In fact, they will have a lower moral status. The higher the forms of humanness that can be realized, the higher one's moral status. From the African perspective, our goal ought to be to realize higher degrees of moral status (Metz, 2012). Suppose the Metaverse fails to afford individuals the opportunity to realize higher forms of humanness. In that case, it seems intuitive that many Africans will prize real-life existence above their virtual existence.

## Considering Objections

This section addresses some potential concerns/objections.

### Risks vs. Benefits of the Metaverse as it Pertains to Relationalism

A critic may contend that the Metaverse aims to profit off human emotions and exploit human desires. In a way, this is a failure to showcase humanity since it involves exploitation. Such exploitation fundamentally fails to honour the value of humanness and communal relationships. This failure is sufficient to undermine its acceptance in Africa.

To justify this claim, the critic could point out that the Metaverse is fuelled by the increasing demand for immersion, leading to a development of a range of online games like Sandbox that simulate the natural world and *are addictive* (Grewe & Gie, 2023). Furthermore, the need for a more immersive environment is partly necessitated by desperate circumstances like the COVID-19 pandemic that caused social restrictions and necessitated a new way of relating through digital replacements/transformation of human activities. However, the creators of the Metaverse are not merely interested in helping alleviate human desperate circumstances. Notably, the Metaverse is a business and will be underlined by a business model. Virtual objects and entities have already been classified by the European Union Intellectual Property Office as Class 9 goods, implying they ought to be treated as digital contents that can be copyrighted by commercial entities (Baker-Brunnbauer, 2022). Whose interest will be served when a business model underlines the Metaverse? Evidently, this will impact the behaviours that are encouraged on the platform, whether (and how) data is collected in the Metaverse and/or shared by commercial entities (or providers) with regulatory bodies. Can commercial entities be trusted to act ethically?

Other issues can arise when commercial entities get involved in the Metaverse. Suppose the boundary between the virtual and physical worlds becomes very blurred, as anticipated by the developers and deployers of the Metaverse. In that case, whoever controls users' information in the Metaverse, could, in fact, be controlling their real lives. The commercial entity could program the algorithm underlying the Metaverse—or establish rules that have significant real-life implications for users. They could determine what information/knowledge users can access or leverage in real life. The preceding also implies that the Metaverse will be built differently for users. In this regard, interactions in the Metaverse can become opportunities to infer users' real-life habits, behaviours, and psyches and control them in real life. It is vital to note that the Metaverse has no private mode. Hence, individuals who use avatars that correspond with their real-life existence risk disclosing personal information that could be used to realize malicious intents. Commercial entities, developers and providers could introduce more subtle forms of control to each user. For example, developers could develop algorithms that spread harmful news. Although the Metaverse promises to be an *open* platform, in reality, it will give developers and commercial entities who invest in the project significant power to spread viral harmful news quickly, suppose they have malicious intent.

In addition to developers, providers and commercial entities behaving unethically, malicious agents can use the system to lure unsuspecting avatars or defraud them. There are reported cases of sexual, verbal and virtual assault by individuals emboldened by these virtual platforms' anonymity (Benrimoh et al., 2022). These assaults on the virtual platforms may become physical, suppose advances in haptic technologies allow individuals to *actually* experience the assaults they suffer in the digital world.

Admittedly, these objections are economic/political ones. However, they have real implications for the claim that the Metaverse is not inherently problematic from the African moral philosophy perspective. In response, the article acknowledges that these risks associated with the Metaverse are possible. Nonetheless, they do not yield the conclusion that the project itself is unethical and should be discouraged on this account, at least from the African moral philosophy perspective. Instead, they point out that the Metaverse may be exploited to realize sinister goals. The preceding point necessitates the significance of establishing norms of ethical/legal behaviours in the Metaverse—within this context—to better reflect African values and foster the project’s uptake in Africa. Importantly, suppose the development and deployment of the Metaverse reflect African core moral values. In that case, these concerns would likely not occur. For example, given the importance of genuinely relating with others and showcasing humanness in this philosophy, exploitation (such as by unethically manipulating others or using data about users to defraud them) will be a failure to wish others well and thus, showcase humanity to them. Admittedly, the preceding sentence does not imply that commercial entities cannot conduct business in the Metaverse. Instead, it implies that this should not be had at the cost of harming or exploiting others. To limit the possibility of exploitation, a norm requiring commercial entities to share profits with users (or benefit sharing) could also be established for the Metaverse. There are many ways to realize this, such as through blockchain technology. A critic could still point out here that although the development of the Metaverse may reflect African moral values. This may not be sufficient to prevent unethical behaviour in the digital world. Unethical behaviours can undermine the uptake of this technology. Suppose the development of this project reflected African moral values and users behaved unethically despite this. In that case, it would not be the failure of the moral philosophy but the failure to act in moral ways as described by the philosophy. Such failures to act in moral ways ought to be identified and punished. Notably, immoral behaviours in the Metaverse can be identified and avatars punished, for example, through reputation-based systems attached to each user, as suggested by Bermejo and Hui (2022).

### The Impact on the Environment

Another critic could point out that the Metaverse's environmental impact will be huge, and this is sufficient to undermine the technology’s uptake in Africa since its deployment (given the environmental impact) will fail to honour African values—for example, those about the environment. To understand how, recall that African view of life tends to be wholistic, implying that communal relationships extend to the environment/nature. However, the energy consumption for the Metaverse will come at a cost to the environment and human relatedness to it. The platform will run on blockchain, which will require high power consumption. Blockchain operates on the consensus algorithm called proof of work, unlike the proof of stake. The former is energy-intensive (Benjamins et al., 2022). In the blockchain, individuals are rewarded based on the amount of mining they perform. The more mining one performs, the higher the probability of being rewarded. What this implies is that individuals would be compelled to do more mining, which would then increase energy consumption. And this will negatively impact the environment, thus worsening current climate change crisis.

In response, it is crucial to clarify how the African moral philosophy conceptualizes a right relationship with the environment. Notably, a right relationship with the environment consists of ecological justice. Precisely, while the thinking about humanness implies that certain ways of showing up in the Metaverse will be immoral, ecological justice means that certain ways of running/operating the Metaverse will be unethical. Concretely, this would imply that more energy-efficient ways of operating the Metaverse should be researched, and the valuable outcomes of such research should be implemented. Moreover, there are reasons to believe that Metaverse will likely reduce human environmental impact. For instance, carbon emissions from vehicular movements and frequent travels by other means have greatly impacted climate change. With the deployment of the Metaverse, individuals would not have to travel long distances to interact with others since interactions would now be had in the Metaverse, thus limiting human movements. This seems to honor the African view of ecological justice.

## Conclusion

This article draws on the moral norms arising from the components of communal interactions and humanness in the scholarship of African moral philosophers to argue that the Metaverse ought to reflect core African values to foster its uptake on the continent. The Metaverse does not yet exist; however, what this technology proposes deserves to be interrogated from multiple perspectives. This article only contributes an African perspective. Implementing norms of acceptable behaviours in the Metaverse will be challenging. Who would have this responsibility? Could a single security apparatus and government or community be established for the Metaverse? What level of influence and control could commercial entities and providers have in the Metaverse? How would behaviours be categorized as unethical? Compelling answers to these questions will require sustained and more elaborate reflection that this article can afford. Hence, the article recommends these questions for future research studies.

## Data Availability

No new data were created or analyzed in this study. Data sharing is not applicable to this article.
